# Implementation of a patient-collected audio recording audit & feedback quality improvement program to prevent contextual error: stakeholder perspective

**DOI:** 10.1186/s12913-021-06921-3

**Published:** 2021-08-30

**Authors:** Sherry L. Ball, Saul J. Weiner, Alan Schwartz, Lisa Altman, Amy Binns-Calvey, Carolyn Chan, Corinna Falck-Ytter, Meghana Frenchman, Bryan Gee, Jeffrey L. Jackson, Neil Jordan, Benjamin Kass, Brendan Kelly, Nasia Safdar, Cecilia Scholcoff, Gunjan Sharma, Soumya Subramaniam, Frances Weaver, Maria Wopat

**Affiliations:** 1 Research Services, Louis Stokes Cleveland VA Medical Center, Cleveland, OH USA; 2 Department of Medicine, University of Illinois at Chicago, Chicago, USA; 3 Center of Innovation for Complex Chronic Healthcare, Jesse Brown VA Chicago Health Care System, Chicago, IL USA; 4 Department of Medical Education, University of Illinois at Chicago, Chicago, USA; 5 Office of Healthcare Transformation, VA Greater Los Angeles Healthcare System, Los Angeles, California USA; 6 Center of Innovation for Complex Chronic Healthcare, Edward Hines Jr VA Hospital, Hines, IL USA; 7 Department of Medicine, Yale University School of Medicine, New Haven, CT USA; 8 Primary Care, Louis Stokes Cleveland VA Medical Center, Cleveland, OH USA; 9 Department of Medicine, VA Greater Los Angeles Healthcare System, Los Angeles, California USA; 10 Department of Medicine, Edward Hines Jr VA Hospital, Hines, IL USA; 11 General Medicine Division, Clement J. Zablocki VA Medical Center, Milwaukee, WI USA; 12 Department of Medicine, Medical College of Wisconsin, Milwaukee, WI USA; 13 Department of Psychiatry and Behavioral Sciences and Preventive Medicine, Northwestern University Feinberg School of Medicine, Chicago, IL USA; 14 Research Services, William S. Middleton Memorial Veterans Hospital, Madison, WI USA; 15 Department of Public Health Sciences, Loyola University Chicago, Chicago, IL USA; 16 Pharmacy Services, William S. Middleton Memorial Veterans Hospital, Madison, WI USA

**Keywords:** Patient-collected audio, Performance improvement, Quality improvement, Contextualization of care, Contextual error

## Abstract

**Background:**

Using patient audio recordings of medical visits to provide clinicians with feedback on their attention to patient life context in care planning can improve health care delivery and outcomes, and reduce costs. However, such an initiative can raise concerns across stakeholders about surveillance, intrusiveness and merit. This study examined the perspectives of patients, physicians and other clinical staff, and facility leaders over 3 years at six sites during the implementation of a patient-collected audio quality improvement program designed to improve patient-centered care in a non-threatening manner and with minimal effort required of patients and clinicians.

**Methods:**

Patients were invited during the first and third year to complete exit surveys when they returned their audio recorders following visits, and clinicians to complete surveys annually. Clinicians were invited to participate in focus groups in the first and third years. Facility leaders were interviewed individually during the last 6 months of the study.

**Results:**

There were a total of 12 focus groups with 89 participants, and 30 leadership interviews. Two hundred fourteen clinicians and 800 patients completed surveys. In a qualitative analysis of focus group data employing NVivo, clinicians initially expressed concerns that the program could be disruptive and/or burdensome, but these diminished with program exposure and were substantially replaced by an appreciation for the value of low stakes constructive feedback. They were also significantly more confident in the value of the intervention in the final year (*p* = .008), more likely to agree that leadership supports continuous improvement of patient care and gives feedback on outcomes (*p* = .02), and at a time that is convenient (*p* = .04). Patients who volunteered sometimes expressed concerns they were “spying” on their doctors, but most saw it as an opportunity to improve care. Leaders were supportive of the program but not yet prepared to commit to funding it exclusively with facility resources.

**Conclusions:**

A patient-collected audio program can be implemented when it is perceived as safe, not disruptive or burdensome, and as contributing to better health care.

**Supplementary Information:**

The online version contains supplementary material available at 10.1186/s12913-021-06921-3.

## Background

Measures of health care provider performance generally rely on information collected from the electronic medical record, claims data, and from patient surveys [1]. These quality measures assess clinician adherence to evidence-based practices and the patient experience [2]. However, effective care also requires a process of patient-centered decision making, termed “contextualizing care,” in which clinicians identify relevant, patient-specific circumstances and behaviors to formulate a contextually appropriate care plan [3]. For instance, a patient presenting with poor control of a chronic condition – such as diabetes, asthma or hypertension -- might hint or directly indicate that they cannot afford their medication, that they are confused about how to take it, or that they are unable to self-manage their medical condition since the departure of a care partner. Each requires a different intervention. In none of these examples would adding more medication be appropriate. And yet a physician who is inattentive to life context could do just that, an oversight that has been termed a “contextual error.” [4, 5] Based on only a medical record review, however, it would appear the physician performed correctly.

Contextual errors are common [6], adversely affect health care outcomes [7], and drive up health care costs [8]. They are detectable by analyzing audio recordings of the medical visit, utilizing a coding method called “Content Coding for Contextualization of Care” (or “4C”) [9]. Based on these research findings, starting in 2012 with two sites and expanding to six, the Department of Veterans Affairs (VA) initiated an audio recording based quality improvement (QI) program to measure and improve clinician attention to patient life context in care planning during ambulatory care visits [10]. Recordings are collected with the assistance of patients who learn about the opportunity in the waiting room where they are invited to carry an audio recorder into their visit and return it to study staff when they leave. Participating patients are instructed that they may carry it out in the open or conceal it, and that they can turn off the device at any point if they change their mind.

Once collected, the recordings are 4C coded to generate de-identified examples of both contextualized care and contextual error, which are then shared by a peer – designated as a clinical champion -- with all participating clinicians at standing meetings for the purpose of helping them improve their care, a process known as “audit & feedback.” [11] Recently the VA funded a multi-site research study to measure both the effectiveness of the QI program and to study its implementation, utilizing an effectiveness-implementation type 2 hybrid design [12]. Over 1000 visits were recorded by patients annually for 3 years across six facilities and analyzed by 4C; outcomes were analyzed 4–6 months post visit using a blinded methodology. The program was associated with significant improvements in health outcomes and, in a budget impact analysis, substantial savings well in excess of costs [13].

In this report we present findings of the study’s investigation of the implementation process. The program successfully reached 666 clinicians, with patients recording 4496 visits, and proved effective [12, 13]. The major implementation challenges were cultural rather than logistical. Handing out audio recorders in the waiting rooms and collecting them when patients leave is technically straightforward and does not interfere with the delivery of patient care. Training audio coders to listen to the recordings, extract data, and generate reports for clinical teams can also be routinized. However, acculturating various stakeholders to a work environment in which interactions may be audio recorded for quality improvement purposes through a novel program is a complex process.

The two sites at which the QI program was first introduced had previously participated in a multi-year study of the methodology [7]. Hence they were already familiar with the program when it transitioned from research to QI. A major difference is that QI, in contrast to research, is generally not optional for clinicians and falls under the oversight of a facility’s quality assurance committee rather than its institutional review board. Its aim is to improve care rather than to advance generalizable knowledge.

In light of the benefits of a patient-collected audio program for improving care and even health care outcomes, there is value in understanding stakeholder perspectives on such an intrusive approach to data collection. Prior studies on the implementation of audio recording at the point of care are predominantly of programs for providing recordings for patients rather that for quality improvement teams [14]. At least three such studies examine staff perceptions, all of them indicating diverging views [15–17]. Concerns were noted about the medicolegal implications of giving patients recordings of visits, disruptions in workflow of an audio-recording program, breaches in patient privacy and skepticism about the benefits of the recordings. Because the audios were being collected for a different audience and for a different purpose, the relevance of the findings is somewhat limited. Nevertheless, the concerns raised about audio recording can be broadly categorized into those pertaining to safety (e.g. liability for provider and data security for patients), disruption (interfering with processes of care), and value (whether it is beneficial to patient care).

Based on these studies, along with lessons-learned during the initial research phase when the program was first developed, the Contextualizing Care Program (CCP), as it is named, was designed around three principles applicable to clinicians, patients, and the facility leadership who control resources [18]: first that it feel safe, second that participating is not disruptive or a burden and, third, that its value is evident to participants. Specifically, clinicians are most likely to feel comfortable participating and remain engaged if: (a) they are confident the findings will never be used punitively and, in fact, are never disclosed in an identifiable form unless they personally request their own data; (b) it will not add additional work to their assigned duties; and (c) they find it helpful. Similarly, patients are most likely to feel comfortable if they are confident the audio recorded data is secure, that participating will not complicate their visit, and that the information collected is utilized exclusively to improve patient care. Finally, facility leadership should regard the program as palatable to staff, not disruptive to care, and as advancing the quality of care.

Hence, each component of the program was designed around these three principles. For example, to optimize security, data are collected on encrypted devices and uploaded to a server accessible only to the coding team and that meets VA standards for the storage of protected health information. To minimize disruption, clinicians simply provide usual care, with no role in the audio recording process, and aggregate feedback occurs during standing meetings. To demonstrate value, feedback incorporates a range of practical examples from audio recordings illustrating the direct link from the physician’s attention to patient life context to patient health care outcomes extracted from the medical record of patients at 4–6 months post recorded and coded encounter. In addition, physicians receive Maintenance of Certification (MOC) credit from the American Board of Internal Medicine for participation, another tangible value. These three principles for the adoption of a patient-collected audio-recording program are further detailed in an earlier publication [10]. Here we examine the extent to which stakeholders did, in fact, perceive the intervention as safe, not disruptive or burdensome, and beneficial to individual providers, patient care and the organization.

## Methods

We utilized mixed methods to gather data for a deductive thematic analysis of stakeholder perceptions of the safety, level of disruption or burden, and value of the patient-collected audio program. Data collection consisted of surveys, focus groups, and structured interviews. In addition, we conducted an inductive thematic analysis of unstructured data collected in the focus groups, the open response components of the survey and the leadership interviews. Subjects consisted of three groups: (a) patients recording their visits, (b) clinicians and other staff whose patient interactions were recorded, and (c) employees with leadership responsibility either within the participating clinics or at the organizational level. This study was approved by the VA Central Institutional Review Board.

The setting consisted of six hospital based ambulatory care clinics within the Department of Veterans Affairs, the United States’ largest integrated healthcare system. Patients were invited to complete a brief anonymous exit survey when they returned their audio-recorder, administered in the third quarter of the first year and the second quarter of the third year. They were asked to respond to three questions -- pertaining to safety, level of disruption or burden, and value -- using a Likert scale: “I felt comfortable recording my visit with my primary care doctor,” “Participation in this project was not disruptive to my interactions today with VA staff,” and “The potential benefits of this project are clear to me.” They were also asked to document anything they liked and disliked about participating and to share any additional comments.

Clinicians were invited to complete an anonymous survey annually, in the third quarter, and to participate in a focus group in the third quarter of the first and third years, the latter to include clinical pharmacists, nurses, and medical assistants who were also occasionally recorded. The survey, which was distributed both in paper form at standing meetings and online via email, included the Organizational Readiness to Change Assessment (ORCA) items, followed by three items with Likert scales assessing perceived safety, level of disruption or burden, and value of participation, and a fourth item measuring self-efficacy (Additional file 1: Appendix A: Provider Survey). ORCA is a validated instrument designed to elicit perceptions of providers, staff, and leadership about readiness to change and perceptions of the evidence, context, and support (facilitation) around implementation [19].

Focus group discussions were led by the local study clinical champion from each of six VA sites. Participants were recruited by email and verbal invitation and provided lunch if they attended. Prior to each focus group an experienced qualitative interviewer and analyst (SLB) from the study team trained the clinical champion by phone on how to facilitate the session. They reviewed a guide designed for the study that included an opening script, the use of grounded prompts designed to draw out participants’ responses on various topics, and specific questions to elicit participant perspective on the principles of safety, disruption or burden, and perceived value of the program (Additional file 1: Appendix B: Focus Group Facilitation Guide). In addition, there were items to assess participants’ opinions about whether the program should be sustained long term. During the actual session, the experienced trainer listened in over a conference line and had the opportunity to ask additional questions as needed. Participants included primary care physicians, clinical pharmacists, nurse practitioners, licensed practical nurses, and an advanced medical support assistant. They were asked not to refer to each other by name and each picked an alias as a substitute. All focus groups were audio-recorded, transcribed, and analyzed. For the analysis, initially a deductive approach was employed, utilizing a priori codes that focused on the three categories of safety, level of disruption or burden, and value of participation. In addition, the two researchers analyzing the data adopted an inductive approach for exploratory purposes supported by qualitative data analysis software, NVivo 12. Consensus building occurred during team meetings and discussions over several months, allowing themes to fully evolve.

Finally, leadership interviews were arranged during the last 6 months of the study with the goal of including five individuals at each of the six participating sites, selected by the clinical champion based on their familiarity with the program. These would all be conducted by the same qualitative interviewer (SLB) who led the focus group training and provided supervision. They were planned as 20–30-min semi-structured phone interviews for which an interview guide was prepared, including an opening script (Additional file 1: Appendix C: Leadership Interview Guide). Participants were initially queried about their level of familiarity with the program and then asked about their perceptions of its safety, level of disruption or burden, and value to patients and providers and to the facility overall. They were also asked to comment on their impression of how the program was implemented, and their interest and readiness in maintaining the program.

## Results

Two hundred fourteen clinicians completed the anonymous survey over the course of the 3 years during which it was offered to a pool of 666 clinicians across the six participating sites. Sixty six, 71, and 77 surveys were completed in 2017, 2018, and 2019, respectively. It is not known how many were yearly repeats, so the participation rate is unknown. Table 1 shows the composition of the 12 clinician focus groups conducted, which included 89 participants across the 6 sites. In addition, 30 leadership interviews were conducted, and 800 Veterans completed surveys (402 in 2017 and 398 in 2019), equally distributed across the 6 sites (66–67 surveys per site).

**Table 1:** Composition of focus groups at each of the 6 study sites

Site code	Focus Group #1 (n) Number present and position		Focus Group #2 (number also present in group #1)	
A	8	4 staff primary care physicians 3 pharmacists 1 registered nurse	10 (3)	2 registered nurses 1 resident 2 pharmacists 5 staff primary care physicians
B	6	6 staff primary care physicians	6 (3)	6 primary care providers
C	7	1 nurse practitioner 4 residents 2 staff primary care physicians	7 (0)	2 LPNs 2 residents 2 staff primary care physicians 1 unknown
D	10	7 staff primary care physicians 2 nurse practitioners 1 pharmacist	6 (5)	5 staff primary care physicians 1 nurse practitioner
E	8	6 staff primary care physicians 1 registered nurse 1 nurse practitioner	7 (0)	2 staff primary care physicians 2 licensed practical nurse 3 clinical pharmacists
F	6	6 staff primary care physicians	8 (0)	3 staff primary care physicians 2 registered nurses 1 licensed practical nurse 1 advance medical support assistant 1 unknown

Perceptions of the extent to which the patient-collected audio program feels safe, not disruptive or burdensome, and valuable followed by other emergent themes related to implementation are presented for each of the three stakeholders: veterans (surveys), clinicians (survey and focus groups), patients (surveys), and facility leaders (structured interviews).

### Safety

**Fig. 1:**
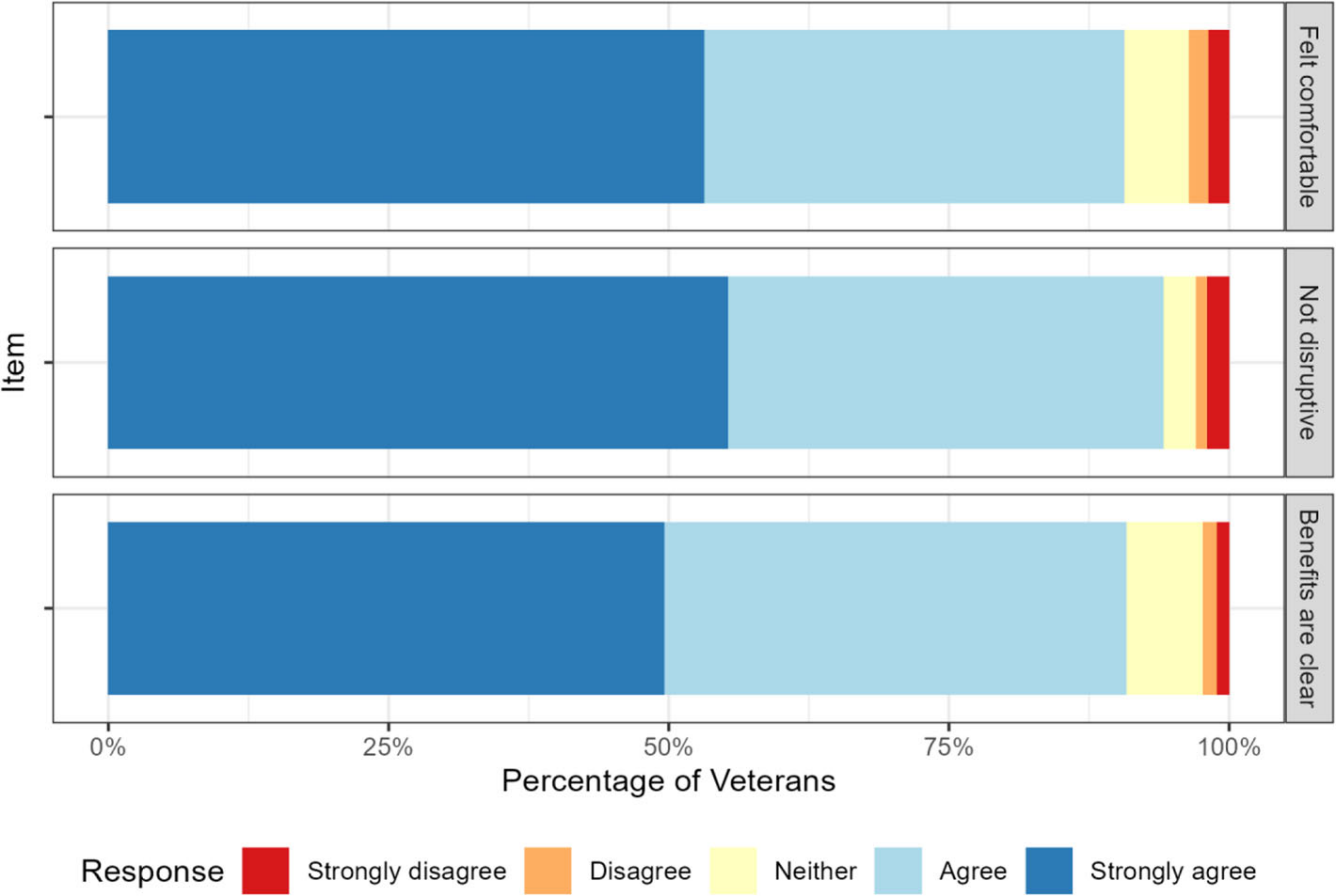
Patient responses on exit survey regarding perceived safety, burden, and value of participation in audio recording program

Veterans and clinicians endorsed the safety of the program. Veteran responses to the safety item “I feel comfortable recording my visit with my doctor” appear in the top panel of Fig. 1; 91% of Veterans agreed or strongly agreed with the statement. There were no significant differences across the sites in responses to the patient items, nor were there significant changes to their responses over the three administrations of the survey.

**Fig. 2:**
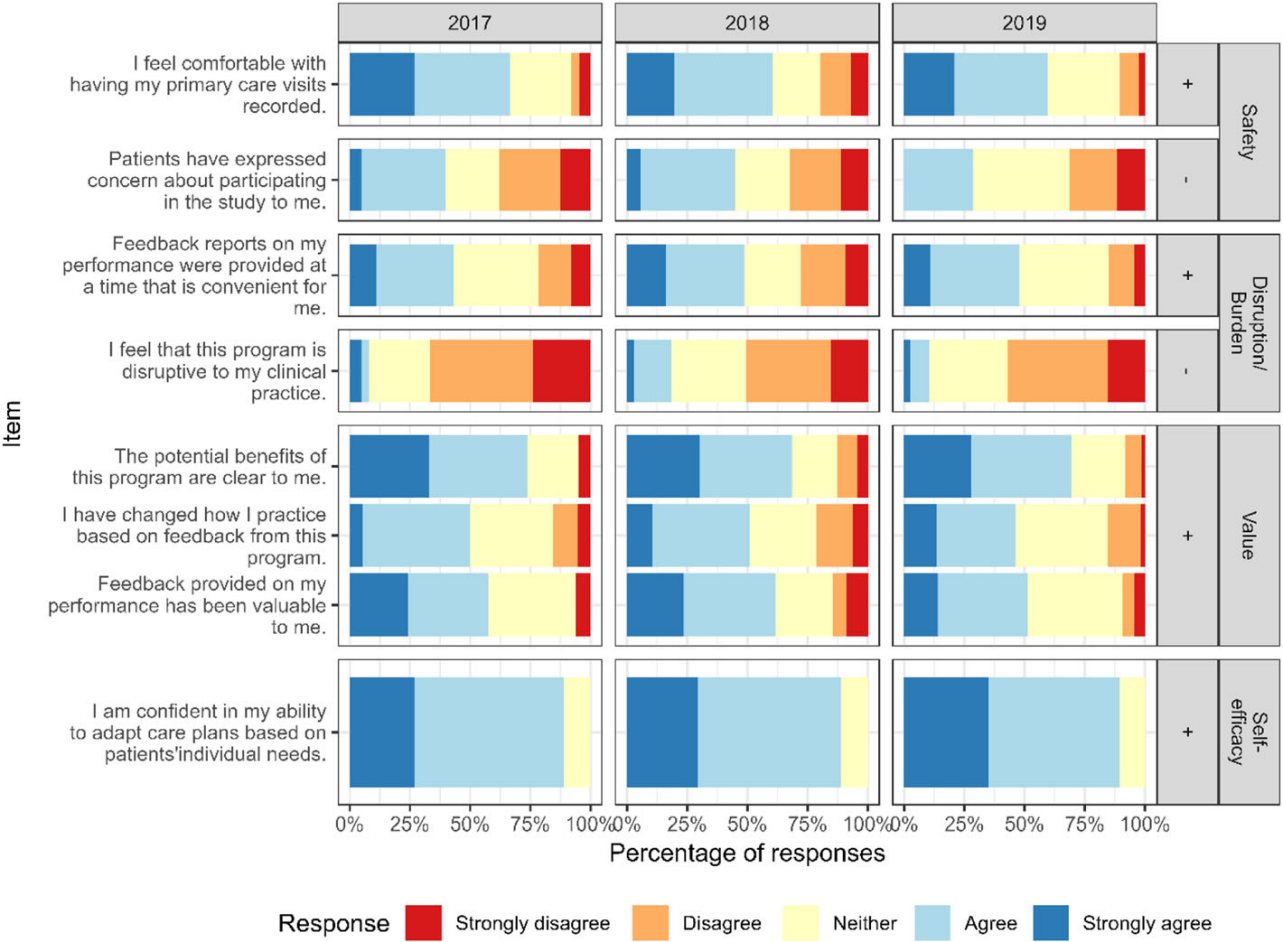
Provider survey responses from 2017 to 2019 to regarding burden, value and safety of the program

Clinician responses to the safety items are shown in the top panel of Fig. 2. Regardless of year, over 62% of clinicians strongly agreed or agreed that they felt comfortable with having been recorded.

In the clinician focus groups, a number of participants reported feeling initial concern about being audio recorded that resolved once they came to see first-hand how the data were utilized. One participant characteristically said, in the first round of interviews, “I think, initially, I was nervous, thinking, ‘Oh, my gosh. What are they going to be doing?’ and everything.” Another expressed a common concern that they “were being graded,” and could be judged if things they said were taken out of context: “I think the hardest part was how honest could I be in a conversation. I had already built up a rapport with most of these patients to be able to talk very frankly with them, and the recorder made it seem like I have to watch what I say.” In the second round of focus groups, comments such as this one were more typical: “I think that I forget that people have recorders. When I’m speaking with patients, it’s usually, I think, how I would normally talk to patients. I don’t think it really changed how I interact, I guess, with patients,” and “I don’t think I’m aware of it until on rare occasion I have a patient bring it up and say, “Oh, I was asked to wear this recorder. Once that happens, I am a little aware of it for a few minutes, but there’s just so much to do and so much to cover that it just quickly goes out of my brain.”

Some providers also expressed concerns about whether patients might be uncomfortable participating even if they volunteered to do so. One put it this way: “The patients don’t fully understand it sometimes, and they feel sometimes -- they feel on the defensive. They feel like the government is recording on their doctor who they like. I think that’s the biggest fear from the patients is they don’t understand it’s voluntary and it’s not punitive, and it’s all for performance improvement.” Others, in contrast, saw the program as an opportunity for patients to feel like they were helping to improve care: “I did have a couple patients who told me they were recording, and they actually seemed to think it was kind of neat. The way that they talked about it was, like, they felt they were in on helping the VA, and they were contributing.”

Leaders, including supervisors and managers, were aware of distrust among some participants, especially early on, who suspected that the program was evaluating them in some way even though they had been told it was not. As one interviewee put it, “I think some of the hesitancy, sometimes by staff, and reluctance, especially in the beginning, was that you’re being recorded … And even though this is not supposed to be punitive, there’s always that concern and kind of defense that goes up that this is gonna be used against you in some way.” One perceived that the distrust was greater among nurses: “I hear no concerns from the physicians. I have heard concerns from nursing staff … They don’t come out and say it directly … I think they -- I think they’re afraid it might be used against them, and could be used in a disciplinary matter.” Another, however, commented that it waned with exposure: “I think once the staff [nurses] started getting those results and they can see that there’s nothing in there that’s personalized or anything like that, it’s very objective, very generalized and everything, … , the way it was presented was really for educational purposes. I think they were feeling more comfortable in doing it.”

### Disruption and burden

Veterans and clinicians also endorsed the lack of disruption involved in participation (second panels, Figs. 1 and 2). 94% of patients agreed or strongly agreed that the program was not disruptive; only 3% disagreed or strongly disagreed.

Overall, 12% of clinicians felt the program could be disruptive, but with the number diminishing over time: The percentage who agreed with the statement that “Feedback reports on my performance were provided at a time that is convenient for me” increased from 2017 to 2019, the only change that was significant (*p* = .04).

Initially, providers often felt they were being judged, which seemed a burden. One participant in the first round of focus groups said “I don’t want to speak for everyone but I feel like the majority of us really thought this was more of how do we do everything for a patient within 30 minutes and that is burdensome and it’s difficult to do.” Another said: “When they [patients] bring up multiple issues, I probably have a hard time saying, ‘Okay, can we not work on this today, I don’t have time,’ because I am being recorded. I expect I have to have an ideal visit, so it probably goes a bit longer than otherwise because of that.”

Most saw the time investment as minimal and self-directed: “… We spend a little more time at staffing meetings, so it takes a little more time from that schedule, and then we get the weekly emails. Those take a little time to look at, but they’re very valuable. I stop to read every one of those emails, so that’s the only impact on my workload.” Another commented: “So many other programs are about documentation and clicking this or doing that reminder and making sure the patient lists have all ‘i’s’ dotted and your ‘t’s’ crossed. This is one of the only programs that don’t require a great deal of paperwork and computer jockey work. In that way, it’s sort of complementary because it lets us work on improving our clinical skills without including all that stuff that adds to your workload.” Finally, some thought the program saved time by encouraging a focus on underlying issues: “I feel like it also saves me a lot of time. For example, a patient is not going to care about their A1c if they have all these barriers. I could speak with them for an hour about why insulin is important when the patient’s needs are clearly not being addressed.”

None of the interviewed leaders expressed concern that the program was a work burden. The closest to this perception came from a few who saw the impetus to encourage staff to identify their patients’ underlying life challenges as time consuming while also seeing it as a net reducer of clinical effort in the long run. As one put it: “Well, certainly these things take more time. But, again, in the short run, they take more time. In the long run, if it serves to create a better treatment plan and get diseases under control, it’s a timesaver because you spend less time focusing on out-of-control situations on future visits. It’s like any good investment. You’ve got to put a little capital in the beginning; but if you have faith in the investment and give it time, it will yield dividends in the end. So, I look at this in the same way.”

### Value

The majority of Veterans and clinicians felt the program had clinical value (third panels, Figs. 1 and 2). Patients were enthusiastic, with 91% agreeing that the benefits were clear to them. About 75% of clinicians agreed that the benefits of the program were clear to them, and over half of clinicians reported that they found the feedback valuable and that it had changed how they practice.

In focus groups, clinicians identified receiving feedback on their attention to patient life context in care planning as a unique experience. As one participant put it, “I think this is one of the areas that we don’t get a lot of feedback on. I mean you can look at notes and at outcomes and all those other things, but there’s nothing like this interaction piece and it’s just something that happens behind closed doors all the time. So how else are you going to know?” Another commented that it sensitized them to their own biases: “Yeah, either you’re tuning out stuff [that patients are saying] because of a bias or you just need to retune your ears. So, to me, the feedback – having enough participation and having enough feedback is, to me, really key because then you start to see your own pattern.” Several also emphasized how important it was that the feedback was facilitated by clinical champions who were respected peers: “I think the important thing is who it’s coming from. Do we actually respect and know the person that the message is coming from, and are they somebody who understands our daily struggle and pleasure within our job and motivations?”

Focus group attendees often mentioned the weekly emails from study staff, which alternate examples in which a clinician addresses an underlying contextual issue with those in which they have not, as especially helpful. One participant commented that “… they just remind me, oh, I need to make sure that I check on something if a similar scenario happened [during one of my visits] so I’m on track. And so, it is more to remind me, to make sure that this happens, and I’m doing more questioning.”

Most but not all leaders we interviewed perceived the program to be valuable. A common sentiment was encapsulated by one leader who said: “I think it’s probably long overdue. It’s the next generation of care. I’ve read this once before that most of the questions in providing clinical care have been answered. It’s really the delivery of that care that’s the biggest gap, and this is one part of that. Almost all of the complaints I get go under communication.” A clinic director commented that it provides a positive means for facilitating improvement in an area that is hard to monitor, saying:

I think it's very easy to become sclerotic in one's practice and to not change, because there's really no good mechanism to do that unless I get a complaint, and then it's the wrong sort of feedback. It's a negative feedback as opposed to a positive review. As a manager, there is no [practical] way to sit in on visits and listen to how people interact with other people. So, this, I think, acts as a non-disciplinary, non-managerial approach to helping people get better in their practice and get that kind of feedback that's really quite useful. I think it's rare to find a primary care provider who gets better on their own without feedback. That's hard to do, and this is a way to do that.

A few others saw the program as actually morale boosting because it’s a break from the usual routines:

It helps them to stay excited and engaged about the day-to-day work life. I mean, the day-to-day work life can be really kind of mundane once you understand the majority of how care is delivered. And then, one size doesn’t fit all for each patient that comes into the room, so to have this sort of information coming back helps you learn how to adjust your communication style for the particular situation or particular patient.

Another way in which the program was thought to have a positive impact on clinicians is in giving full agency to participants:

I think what’s nice about the project is you really leave it up to the providers to decide how they want to implement it, so it’s not a mandate where now [they] have to document.

Some saw value in the program because they thought it would reduce unnecessary resource consumption: “If you don’t communicate well with patients, you end up wasting labs and ordering things you don’t need and bringing people back more often.” Conversely, there were others who expressed skepticism about its efficacy: “And as far as the value, I can’t speak to what people are specifically told, but I haven’t seen anything that was identified as in terms of improvement. And giving feedback based on findings doesn’t necessarily lead to improvement or change, so I haven’t seen any data in regards to that.”

### Readiness and sustainability

**Fig. 3:**
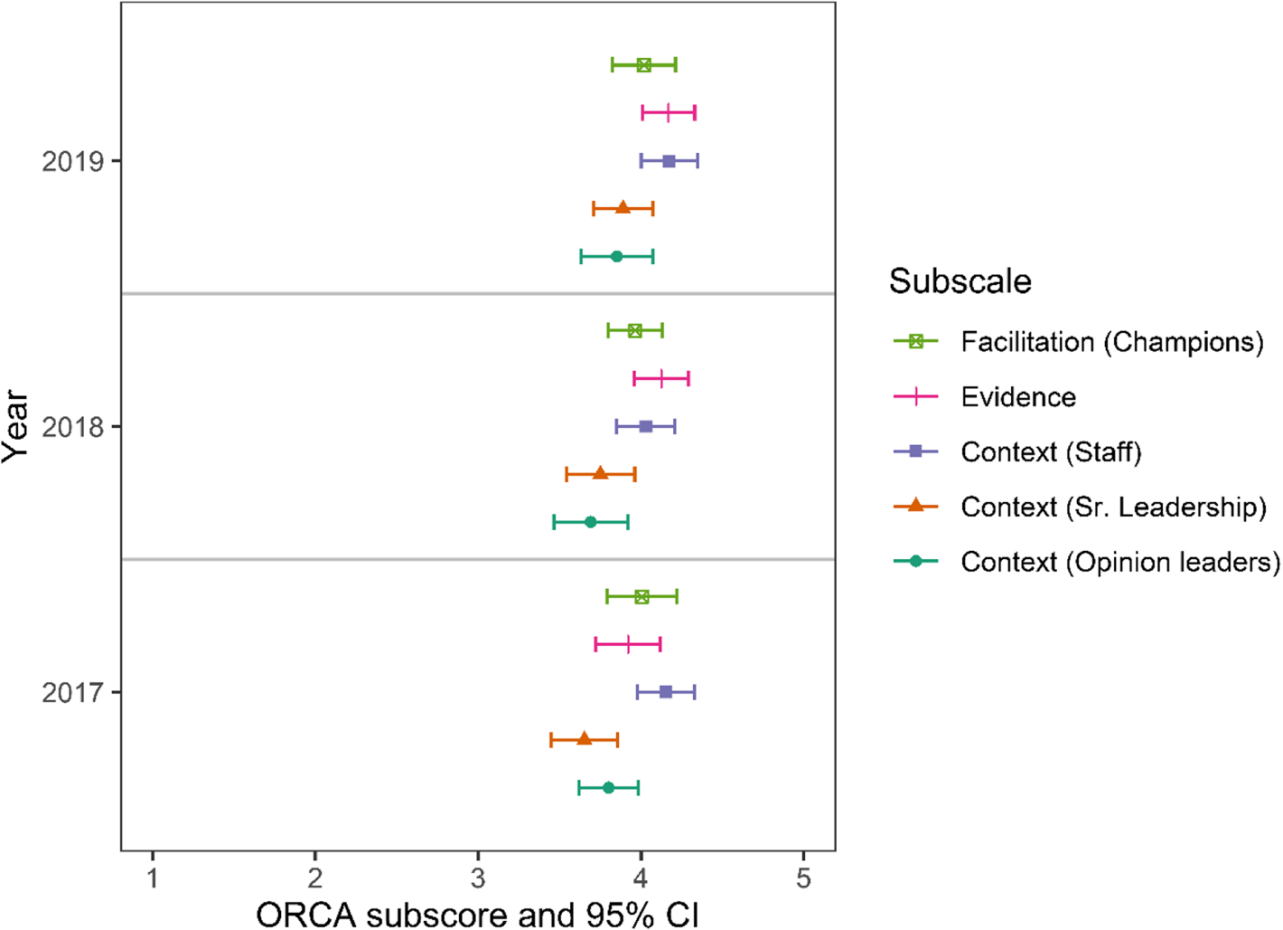
ORCA mean scores with 95% confidence intervals by subscale

Surveyed clinicians agreed that they were confident in their ability to contextualize care in each year (Fig. 2, bottom panel). The ORCA items showed evidence of increasing organizational readiness to change over time (Fig. 3). Clinicians rated the evidence as significantly higher for the statement “The practice of contextualizing care, defined as adapting care plans to patients’ individual needs, results in better health care outcomes” from “very weak” to “very strong” in 2019 compared to 2017 (*p* = .008). They also rated senior leadership and clinical management more highly in 2019 compared to 2017 for overall commitment to quality improvement, based on their responses to 5 ORCA context items, including “provide effective management for continuous improvement of patient care,” and “provide staff members with feedback/data on effects of clinical decisions” (*p* = .02).

Focus group participants commented on whether the program should continue. A common theme was that if the program were discontinued, its benefits could wane. One participant commented that “I have developed the habit [of contextualizing care] already, and that’s a good thing, but reminders help to keep that happening, and not slipping back into bad habits, old habits.” Another participant felt the program might have lasting benefits even if ended:

I think we’ve been participants in the program long enough and getting feedback about it that it’s become habituated in our practices. That being said, if there were no more reminders of this program, would behaviors extinguish over time without that nurturing and cultivation? I don’t know. I think it’s possible. But for now, I think it’s been so long that it’s built into my practice much like learning any other clinical skill. Once you do it enough over time, it becomes pretty routine for you.

Similarly, leaders often articulated that they thought the benefits of the program would be lost if it were not sustained. One interviewee said, “What is monitored tends to get improved. And then once you stop monitoring and you stop observing a practice, it tends to regress back to the mean. So, I do think there would be some value to continuing the project definitely.” Another suggested it should be required, suggesting that they could “make it sort of a condition of employment that you’ll participate and get this feedback as part of helping you professionally develop.”

Nevertheless, despite an appreciation for the value of the program, few felt they could self-fund the initiative if it fell upon their facilities to do so. As one medical center leader put it, “It’s a question of do we have the staff availability, and do we have the resources to have staff manning the desk. It’s resource intensive and money intensive. So, as long as it’s being provided, we probably likely will continue. But if it were to stop and if we were asked to implement, I don’t know we’ll have the funds or the staff to implement it and continue going.” And another indicated they’d like to see further evidence of the value of the program: “We live in a resource-constrained environment, and any resources that are put towards things that are ineffective are wasted resources. So, if this doesn’t improve, I would assume, reduction of contextual errors, then putting resources towards continuing it would not be a good use of resources.”

### Additional veteran reflections

In their survey, Veterans were also invited to respond to three open-ended questions. There were 425 responses to “What did you *like* about participating in the intervention?”, 165 to “What did you *dislike* about participating in the intervention?”, and 70 to “Do you have any *additional comments* about your participation in the project?” Broadly speaking, the responses supported the ratings of safety, lack of disruption or burden, and value, but also included several other notable opinions.

The most common response to the first question could be described as altruistic, as in “I appreciate helping to improve care,” or “Just like the fact that my doctor will have feedback.” Many perceived that their doctor was more thorough when aware the visit was being recorded, as in “my doctor was more on her toes.” Finally, quite a few commented on ease of participation, as in “Frankly, I was not thinking about it [i.e., the audio recording]. I hit play and it didn’t cross my mind.” For the second item, responses were largely assertions that there was “nothing to dislike” about participating. All but 11 fell into this category. Among these 11 were concerns about surveillance, as in “Even though it’s not spying, still feels like it is,” concerns about privacy, as in “Felt nervous talking about sex,” and concerns about impacting the interaction, as in “Felt that it could possibly interfere with doctor-patient communication.” For the third item – additional comments – most responded simply that they had “none” or wrote “all good.” Others were affirmations of support for the program, as in “If this program improves things for other vets as well, it is well worth it,” and “I am glad the VA cares so much about quality care to have a survey like this.” A few had suggestions about the process, sometimes with opposing perspectives, including “I think the doctor should be told who is recording them and who is not” and “I wonder if this program would be more valuable if doctors didn’t know they were being recorded.”

## Discussion

Inviting patients to audio record their visits for the purpose of collecting data to improve care is generally accepted and even embraced if the process is regarded as safe, not disruptive or burdensome, and worthwhile. For physicians and other health care staff, a program feels safe when they trust that their audio recorded interactions will not be used punitively and, furthermore, that they won’t be shared with identifiers with anyone other than themselves; for patients, it’s when they are confident that their privacy is not compromised and, in addition, that they are not unwittingly enlisting in surveillance of their physicians. Not disruptive or burdensome means that participating clinicians are not required to do additional paperwork or attend additional activities as a result of the program, and that it is left up to them to decide how and whether to alter their care, with no consequences if they don’t; for patients, it’s that participating doesn’t distract them from their visit. And, finally, worthwhile (or valuable) means that clinicians see first-hand that the feedback they obtain is eye-opening, convincing, and practical. Receiving needed credits towards board re-certification and/or maintaining licensure is also a value. For patients, valuing the program entails understanding enough about it to appreciate that they may be improving health care by participating.

Based on data collected from the focus groups and leadership interviews, it appears that perceptions about the program’s safety increased over the course of the study as participants came to see and trust that the data were only used for its intended purpose. That a respected peer served as clinical champion may have been a reassuring signal that the initiative was credible as well as worthwhile. Notably, however, the physician survey did not identify any measurable increase in perceptions that the program was safe, although it did document a relatively highly level of baseline level of comfort with having visits recorded. That participation in the survey was entirely voluntary and, hence, non-representative, and that those who participated in the second round were not necessarily the same as those who participated at the start of the study, may account for inconsistencies between the quantitative and qualitative findings.

Perceptions that the program is disruptive or burdensome were uncommon, with just 12% of clinicians surveyed and 3% of patients expressing this concern. Furthermore, there was a significant increase among the former with regard to finding the feedback process “convenient.” Perceptions that the program is valuable also increased among clinicians significantly, as reflected in their increased confidence that it was based on strong evidence. And over 90% of patients reported that “the potential benefits of this project are clear to me.”

Finally, facility leaders were supportive of the program, mirroring the perceptions of providers. Few saw it as an impediment to health care delivery. Some even regarded it as a morale booster and a stand-out from other quality improvement initiatives because it appeals to clinicians’ and other staffs’ self-motivation to improve based on low-stakes feedback rather than employing a carrot or stick. Most also saw the program as inherently valuable, although a few raised questions about the evidence that the feedback would change clinician practice. Despite appreciating the program, however, few indicated they would or could fund the program with local resources exclusively.

A limitation is that the findings may not generalize to settings outside the Veterans Health Administration. Also, participants in focus groups and surveys may not be representative; they could be either more or less likely to feel positively about the program. Finally, we do not yet know the extent to which the program will be maintained – only that it garnered the support of key stakeholders. Sustaining it will require long term investment for an initiative that is not mandated. The fact that a program that requires audio-recording medical encounters is seen as safe, not disruptive or burdensome, and worthwhile may be necessary but not sufficient to sustain it. At the time of the study’s conclusion, support for the program varied by site. It had received funding at one new site, and at least one existing site had committed to continued funding of the program. All activity ceased with the onset of the COVID-19 pandemic.

## Conclusions

A patient-collected audio program that adheres to principles of safety, avoidance of work disruption or burden, and for which there is a demonstrated evidence base can be effectively introduced in a health care setting with the support of clinical staff, patients, and facility leaders. Although this study examined the implementation of a program to improve the attention of physicians and other health professional to patient life context in care planning, it may provide generalizable information about how to implement patient-collected audio recording for other quality improvement purposes. The physician-patient encounter has been called an impenetrable “black box” as physician and patient interact behind closed doors, with assessments relying entirely on secondary data from the medical record or reports by patients of the experience [20]. Patient collected audio offers a viable strategy for directly observing care.

## Supplementary Information

**Additional file 1: Appendix A**: Provider Survey with ORCA Questions. **Appendix B**: Focus Group Leader Facilitation Guide. **Appendix C**: Leadership Interview Guide.

## Data Availability

The datasets used during the current study are available from the corresponding author on request if approved by the Department of Veterans Affairs through a Data Sharing Agreement.

## Abbreviations

4C: Content Coding for Contextualization of Care
ORCA: Organizational Readiness to Change Assessment
QI: Quality improvement
VA: Department of Veterans Affairs
