# Supplementary material for: Implementation of a patient-collected audio recording audit & feedback quality improvement program to prevent contextual error: stakeholder perspective
Source: BMC Health Serv Res. 2021 Aug 30;21:891. doi: 10.1186/s12913-021-06921-3 (PMC8403819; doi:10.1186/s12913-021-06921-3)
Supplement: Supplementary file 1 — Additional file 1: Appendix A: Provider Survey with ORCA Questions. Appendix B: Focus Group Leader Facilitation Guide. Appendix C: Leadership Interview Guide. [file 12913_2021_6921_MOESM1_ESM.pdf]

Appendix A
Provider Survey with ORCA Questions

Organizational Readiness to Change Assessment

I. Evidence Assessment

Findings statement: The practice of contextualizing care, defined as adapting care plans to patients' individual needs, results in better health care outcomes.

	Very weak	weak	Neither weak nor strong	strong	Very strong	Don't know/ Not Applicable
a) Based on your assessment of the evidence* for the above **findings statement,** rate the strength of evidence:	1	2	3	4	5	NA
b) Now, rate the strength of evidence for this statement based on how you think your ***colleagues*** at your local VA feel about the strength of evidence:	1	2	3	4	5	NA

*Evidence includes data, reports and information from presentations provided to you at PACT team meetings, emails with cases on the topic, published articles etc….

II. Context Assessment

For each of the following statements, please rate the strength of your agreement with the statement, from 1 (strongly disagree) to 5 (strongly agree).

Staff members at your facility:	strongly disagree	disagree	neither agree nor disagree	agree	strongly agree	Don't know/ Not Applicable
a) have a sense of personal responsibility for improving patient care and outcomes	1	2	3	4	5	NA
b) cooperate to maintain and improve effectiveness of patient care	1	2	3	4	5	NA
c) are willing to innovate and/or experiment to improve clinical procedures	1	2	3	4	5	NA
d) are receptive to change in clinical processes	1	2	3	4	5	NA

Senior leadership/clinical management at your facility:	strongly disagree	disagree	neither agree nor disagree	agree	strongly agree	Don't know/ Not Applicable
a) provide effective management for continuous improvement of patient care	1	2	3	4	5	NA
b) clearly define areas of responsibility and authority for staff	1	2	3	4	5	NA
c) promote team building to solve clinical care problems	1	2	3	4	5	NA
d) provide staff members with feedback/data on effects of clinical decisions	1	2	3	4	5	NA
e) reward clinical innovation to improve patient care	1	2	3	4	5	NA

Opinion leaders at your facility:	strongly disagree	disagree	neither agree nor disagree	agree	strongly agree	Don't know/ Not Applicable
a) encourage changes in practice patterns to improve patient care	1	2	3	4	5	NA
b) support changes in practice patterns to improve patient care	1	2	3	4	5	NA
c) are willing to try new clinical protocols	1	2	3	4	5	NA
d) work cooperatively with senior leadership/clinical management to make appropriate changes	1	2	3	4	5	NA

III. Facilitation Assessment:

For each of the following statements, please rate the strength of your agreement with the statement, from 1 (stongly disagree) to 5 (strongly agree):

The program clinical champions*:	strongly disagree	disagree	neither agree nor disagree	agree	strongly agree	Don't know/ Not Applicable
a) accept responsibility for the success of this program	1	2	3	4	5	NA
b) have the authority to carry out the implementation	1	2	3	4	5	NA
c) are considered a clinical opinion leader	1	2	3	4	5	NA
d) work well with the intervention team and providers	1	2	3	4	5	NA

*

Assessment of Principles Guiding the Contextualizing Care Program

These final questions are about your perceptions of the program and about your participation experience. Please indicate the extent to which you agree or disagree with the following statements.

Principle: Safety

I feel comfortable with having my primary care visits recorded.

Strongly Disagree   Disagree   Neutral   Agree   Strongly Agree

Patients have expressed concern about participating in the study to me.

Strongly Disagree   Disagree   Neutral   Agree   Strongly Agree

Principle: Embedded in Ongoing Activities

I feel that this program is disruptive to my clinical practice.

Strongly Disagree   Disagree   Neutral   Agree   Strongly Agree

Feedback reports on my performance were provided at a time that is convenient for me.

Strongly Disagree   Disagree   Neutral   Agree   Strongly Agree   NA

Principle: Value of Program Evident to Providers

The potential benefits of this program are clear to me.

Strongly Disagree   Disagree   Neutral   Agree   Strongly Agree   NA

Feedback provided on my performance has been valuable to me.

Strongly Disagree   Disagree   Neutral   Agree   Strongly Agree   NA

Principle: Self-Efficacy

I am confident in my ability to adapt care plans based on patients'individual needs.

Strongly Disagree   Disagree   Neutral   Agree   Strongly Agree

I have changed how I practice based on feedback from this program.

Strongly Disagree   Disagree   Neutral   Agree   Strongly Agree   NA

Finally, do you have any additional comments about your participation in this program? If you'd like to expand upon any of the answers you provided above, you can do so here.

____

____

____

____

____

____

Thank you for your participation in this study and your very valuable feedback!

Appendix B
Focus Group Leader Facilitation Guide

Instructions from facilitator: We'd like to talk to you today about your experiences participating in the Contextualizing Care quality improvement program that has been occurring in your facility. Our goal is to understand what your perceptions of the program are so we can use them to ensure that the program is following the core principles including that it is safe, not burdensome, and is valuable to you, your colleagues and patients. Some of these questions may sound familiar. We've asked similar questions throughout the program but are interested in final impressions as well as the potential for continuation of the program. Although we will not transcribe names, you are welcome to use position titles or an alias rather than proper names when referring to yourself or others.

Grounded prompts: If responses are limited or require clarification, probes may be used to elicit more detailed responses. Probes should use verbatim words or phrases presented by the participant using one of the following formats:

1. *What do you mean by ____________?*
2. *Tell me more about ____________?*
3. *Give me an example of ____________?*
4. *Tell me about a time when ____________?*
5. *Who ____________?*
6. *When did/was ____________?*

Principle: Safety

- How do you feel about having some of your visits recorded?
- What concerns, if any, do you have about safety while participating in this program? What can we do to address these concerns for the future?
- What concerns, if any, do you have about the safety of this program for patients who participate? What can we do to address these concerns?
- What concerns, if any, do you have about the safety or security of your patient's private information while participating in this program? What can we do to address these concerns?

Principle: Embedded in Ongoing Activities

- In what ways, if any, does knowing you might be recorded incline you to act differently with your patients?
- In what ways, if any, is this program disruptive to your clinical practice? What can we do to minimize disruption to your clinical practice?
- In what ways, if any, does the program affect your workload? What can we do to minimize impact on your workload?
- In what ways does this program fit with other efforts to promote good clinical practice?

Principle: Value of Program Evident to Patients

- What is your perception of the evidence supporting this program?
- What is your understanding of the potential benefits of this program?
    - o What types of addition information do you think would have been helpful prior to participation in this program? [*Implementation*]
- In what ways do you think this program has improved the care you provide?

    - In what ways do you think this program has improved the way you communicate with patients?
    -
- In what ways has it improved the attention you bring to the challenges in your patients' lives that are relevant to their achieving their health goals?

- In what ways has it improved your ability to set realistic goals for your patients based on their personal circumstances?

Barriers/Facilitators

- What barriers or challenges has this program encountered? What barriers or challenges to you think you might encounter in the future?
- What has facilitated or helped this program be successful at your facility? What might facilitate or help this program going forward?

Added to second round of focus groups

What is your sense that changes to your behavior as a result of the Contextualizing Care program will sustain over time?

What is your sense that the Contextualizing Care program could become part of your facility's normal practice for evaluating care? How do you feel about that?

What are your thoughts on the potential for using the Contextualizing Care approach in other areas of the hospital?

Do you have any additional comments about this study?

Appendix C

Leadership Interview Guide

Interviewer Name: VA site: Position/Role:

Interview Date: Start time: End time:

Hello [Dr. Ms/Mr participant's name],

My name is [ ] and I am the implementation specialist for the contextualizing care program. As you recall, this is a program in which Veterans volunteer to audio record their visits so that the data can be used to give providers with feedback on their performance at addressing the life challenges Veterans face that are relevant to their care. The purpose of the program is to facilitate more patient centered care that focuses on Veterans psychosocial needs.

I am talking to leadership at each of six sites where this program has been implemented. [site PI] is the PI for this program at your site. Information from this interview will be used to improve this program. If you do not feel familiar enough with the program to answer a specific question, please just let me know and we'll move on.

The call will take approximately 20-30 minutes.

Your participation is voluntary. You can stop the interview at any time.

Do you have any questions?

In order to make sure we capture all of the information you give us, we would like to record this call. The audio-file for the recording will be stored directly to restrict access to the file on the VA intranet. Is this okay with you? **[Hit record button.]** Okay, to confirm, I'm starting the recording. Is this ok with you?

Grounded prompts: If responses are limited or require clarification, probes may be used to elicit more detailed responses. Probes should use words or phrases presented by the participant using one of the following formats:
1. What do you mean by ____________?
2. Can you tell me more about ____________?
3. Give me an example of ____________?
4. Tell me about a time when ____________?

Questions for Brief Leadership Interview

- Based on your knowledge of the project, what are your perceptions of the "Contextualizing Care Program?"
  - o How familiar are you with the program?
  - o What are your thoughts about the focus on primary care as the target audience for the Contextualizing Care program?

Next, we'd like to discuss your perceptions of implementation of the Contextualizing Care Program, including your thoughts on how the project adhered to its three core principles which are that it should feel safe, should be embedded seamlessly into ongoing clinic and facility activities, and that the value of the program should be evident to participants.

The first core principle is that the project is safe for clinicians/staff and patients and that they should also feel safe, particularly with regard to confidentiality.

- What concerns, if any, did staff and clinicians, including yourself, have about this project? Were steps taken to mitigate these concerns effective? If not, what are your thoughts on what we could have done better?

- What concerns did patients have about this project? Were steps taken to mitigate these concerns effective? If not, what are your thoughts on what we could have done better?

The second core principle is that the project should be embedded in ongoing activities. The activities of the project should not interfere with regular clinical practice in any way.

- When were the data produced during this study discussed? Was this a good time? What might have been the most effective and least disruptive time for discussion of these data, if any?

- What issues, if any, were there with recruitment of Veterans for the audio-recordings? Do you feel this project affected the flow of care? If so, do you have suggestions or thoughts on how Veterans should be recruited?

The third core principle is that the project's value should be evident to all participants including leadership.

- In what ways did you see the value of the project presented? Can you comment on the effectiveness of this? What thoughts do you have on things we can do to ensure that the value of the project is evident to all participants – clinicians, staff, patients, and leadership?

Finally, we'd like to hear your thoughts on how the program was implemented at your facility. Your responses may be used to inform our efforts moving forward.

- What facilitated the adoption or rollout of this program?
    - What remaining concerns do you have about the intervention, if any?

- From a leadership standpoint what barriers were encountered while implementing this program, if any? What steps, if any, did you take to support implementation?

- What value do you think there could be in maintaining this program after the project is concluded? Do you plan to continue this work in your facility?
    - What potential do you see for implementing the Contextualizing Care Program in other parts of the hospital or in other services? Where do you think it might be most appropriate and beneficial?

- Regardless of your plans to continue or not, what resources do you think would be needed if you were to continue to support the use of this program in your clinic? What adaptations do you think might be needed to maintain this program at your facility?

Thank you for your participation!
